# Leveraging multi-modal data for early prediction of severity in forced transmission outages with hierarchical spatiotemporal multiplex networks

**DOI:** 10.1371/journal.pone.0326752

**Published:** 2025-06-25

**Authors:** Rafaa Aljurbua, Jumanah Alshehri, Shelly Gupta, Abdulrahman Alharbi, Zoran Obradovic

**Affiliations:** 1 Center for Data Analytics and Biomedical Informatics, Computer and Information Science Department, Temple University, Philadelphia, Pennsylvania, United States of America; 2 Department of Computer Science, College of Computer, Qassim University, Buraydah, Saudi Arabia; 3 College of Business Administration, Imam Abdulrahman bin Faisal University, Dammam, Saudi Arabia; 4 Department of Computer Science, College of Engineering and Computer Science, Jazan University, Jazan, Saudi Arabia; Aalto University, FINLAND

## Abstract

Extended power transmission outages caused by weather events can significantly impact the economy, infrastructure, and residents’ quality of life in affected regions. One of the challenges is providing early, accurate warnings for these disruptions. To address this challenge, we introduce HMN-RTS, a hierarchical multiplex network designed to predict the duration of a forced transmission outage by leveraging a multi-modal approach. We investigate outage duration prediction over two years at the county level, focusing on the states of the Pacific Northwest region, including Idaho, California, Montana, Washington, and Oregon. The multiplex network layers collect diverse data sources, including information about power outages, weather data, weather forecasts, lightning, land cover, transmission lines, and social media. Our findings demonstrate that this approach enhances the accuracy of predicting power outage duration. The HMN-RTS model improves 3 hours ahead outage predictions, achieving a macro F1 score of 0.79 compared to the best alternative of 0.73 for a five-class classification. The HMN-RTS model provides valuable predictions of outage duration across multiple time horizons and seasons, enabling grid operators to implement timely outage mitigation strategies. Overall, the results underscore the HMN-RTS model’s capability to deliver early and practical risk assessments.

## 1 Introduction

Adverse weather events, such as thunderstorms and freezing rain, can severely disrupt power systems, resulting in critical failures, compromised quality of life, and significant financial losses [1]. Historically, research on power outages has centered on statistical methods, including Bayesian additive tree models and regression learning [2]. Several studies have specifically investigated outage durations, applying traditional machine learning approaches such as random forests and Bayesian deep learning to the KSTAR disruption database while identifying potential precursors [3–7]. In addition, researchers have explored natural factors (for example, rainfall) that influence outage duration [8], with some employing adaptive ensemble techniques. For instance, one study evaluated various grid-hardening strategies by developing a hybrid mechanistic power-outage learning model [9]. Phasor Measurement Unit (PMU) data has also been a key resource for analyzing power outages [10], and graph-theoretic frameworks have been leveraged to enhance outage predictions [11,12]. However, many of these methodologies rely on a single data source, restricting their scope. To address this limitation, we integrate multiple data from multiple sources such as vegetation, weather, and social sensors.

Power outage predictions heavily rely on weather data, often becoming incomplete during extreme weather events. While previous research explored various methods to address this issue, the potential advantages of integrating noisy weather data with insights drawn from social media posts remain unexplored. Social media platforms have increasingly become central to communication in society [13,14], especially for disseminating urgent information during severe weather situations [15]. Studies show that weather conditions significantly impact social media activity [16]. Social media data can offer valuable insights into the effects of weather on infrastructure and human behavior while providing real-time feedback to those monitoring the situation [17]. Furthermore, research indicates a correlation between the volume of weather-related tweets and prevailing weather conditions [18]. Therefore, merging social media data with traditional weather sensor data can enhance predictions of power outages during extreme weather events.

Predicting power outages is critical, but it is equally essential to forecast the duration of these outages to ensure adequate response and mitigation strategies. Various statistical techniques have been used to estimate the duration of power outages, such as Cox proportional hazards regression and multivariate adaptive regression [19]. However, these methods are limited in their ability to capture the dynamic and seasonal variations that may influence outage duration, as well as their reliance on simplified assumptions about the underlying data. In the era of machine learning, several studies have utilized machine learning models [20], including Random Forest [21] and Extreme Gradient Boosting [22], to predict power outage durations. However, the potential advantages of multiplex networks that integrate data from multiple sources to predict outage durations remain largely unexplored. In our previous work [23], we demonstrated that multiplex networks effectively predict the occurrence of power outages. In our follow-up study [24], we developed a spatiotemporal multiplex network model to predict forced power outages in distribution grids. While this work advances outage prediction using multiplex structures, it did not adopt a hierarchical modeling approach (outage occurrence followed by outage severity) or incorporate social media information. However, our other study [25], which successfully applied multiplex networks to predict power outage duration, did not investigate seasonal variations or assess the relative value of the multiplex network compared to a single-layer model.

In this work, we present HMN-RTS, a hierarchical spatiotemporal multiplex network that integrates structured data, such as weather, land cover, and transmission line details, with unstructured data from social sensors collected over time and space. HMN-RTS predicts power outage durations up to three hours in advance by capturing both environmental conditions and changes in social media activity. Designed to provide early warnings of outage risks, the model has the potential to enhance outage management and response efforts at the county level across the U.S. Pacific Northwest.

We incorporate weather forecast data to build a predictive forecast layer and conduct a detailed analysis of seasonal patterns in outage prediction across the region. To evaluate the advantages of our multiplex network over traditional single-layer models, we conduct a comparative experiment focused on outage severity prediction. Furthermore, we enhance the model architecture, resulting in improved performance relative to baseline approaches. The main contributions of this paper include the following:

1. Enhance the predictive model architecture to improve the accuracy of forced power outage predictions.
2. Conduct detailed Hierarchical Spatiotemporal Multiplex Network analysis to gain an in-depth understanding of the network.
3. Investigate the effectiveness of the multiplex network compare to a single-layer model.
4. Integrate weather forecast as an additional data source and construct a forecast layer to examine seasonal predictions at the county level across the U.S. Pacific Northwest region.

## 2 Related work

The related work for the proposed approach can be summarized into four key components: 1) predicting the duration of power outages, 2) managing missing and incomplete weather data, 3) the significance of the multi-modal approach, and 4) the development of a hierarchical spatiotemporal multiplex network combined with multi-modal data for predicting power outage durations.

### 2.1 Weather data

The Automated Surface Observing Systems (ASOS) ceilometer measures clouds up to 12,000 feet (3.6 km). However, it may compromise the accuracy of ASOS data due to incomplete atmospheric coverage, leading to missing data [26]. Missing data is also a common challenge resulting from sensor malfunctions and cloud contamination [27]. Several strategies have been explored for managing missing data. For example, employing regression-based estimation [28] or using multiple imputations to replace missing data [29]. Other common methods apply mean imputation [30]. However, it is crucial to handle missing data carefully to ensure an accurate analysis, as ignoring missing instances can lead to significant risks in the analysis. Further, the hybrid-triggered dynamic-consensus control for DC microgrids [31] optimizes data efficiency, which is relevant for improving system performance in predicting power outage durations with diverse data sources.

### 2.2 Multi-modal approach

Combining multiple forecasts often outperforms the accuracy of the best individual forecast [32], while ensemble forecasting also provides valuable reliability assessments for predictions [33]. Building on these advancements, recent research has focused on enhancing power outage prediction by employing the multi-modal approach. For example, [34] proposed an innovative approach utilizing multi-level data, including weather observations and forecast information. Similarly, [35] examined the impact of forecast uncertainties on outage predictions, highlighting the significant roles of precipitation and wind gusts as key factors. In addition, [36] studied the effect of lightning features on the prediction of thunderstorm outages.

### 2.3 Power outage duration prediction

For utility companies, predicting power outage duration early and accurately is essential for planning power restoration more effectively. In recent years, researchers have increasingly turned to machine learning models to predict the duration of power outages. For instance, a Random Forest-based model is used to predict the duration of hurricane-related power outages using variables like wind speed and wind duration [21]. Similarly, the duration of outages during typhoon disasters has been predicted by integrating models like Extra Tree (ET) [22], Extreme Gradient Boosting (XGBoost), Light Gradient Boosting Machine (LightGBM), Random Forest (RF), Gradient Boosting Regression (GBR), and Decision Tree (DT). Before the advent of machine learning, statistical methods were commonly employed to predict power outage durations. These methods include accelerated failure time regression, Cox proportional hazards regression, Bayesian additive regression trees, regression trees, and multivariate adaptive regression splines [19]. Furthermore, combining statistical methods with geographic information systems (GIS) has proven effective in analyzing the performance of storm-affected urban distribution systems. For example, one study used GIS to plot data on repair crews during winter storms to study the duration of outages [37]. Other studies have applied Accelerated Failure Time (AFT) and Cox Proportional Hazard (CPH) models to estimate the duration of storm-caused power outages [38]. Night Time Lights (NTL) imagery data has also been utilized to assess outage duration, particularly in Puerto Rico [39]. In addition to weather-related factors, socioeconomic elements have been shown to influence outage duration [40,41].

### 2.4 Multi-modal learning in a hierarchical spatiotemporal multiplex network for predicting power outage duration

Weather data plays a crucial role in predicting power outages [42,43]. However, the high frequency of missing values during severe weather events complicates learning. Our research integrates weather data with social sensor data to address this challenge. We explore the impact of social networks (multiplex networks) and social sensor data on predicting power outage durations. The network structure is crucial for capturing complex interdependencies between different data sources, thereby enhancing the accuracy and robustness of predictions [44–47]. Similar hierarchical frameworks have demonstrated resilience under communication faults, such as in microgrid frequency regulation under PMU failures [48]. Our study is among the first to predict power outage durations three hours in advance by incorporating social sensors within a multiplex network framework. Specifically, we evaluate the benefits of learning from a spatiotemporal multiplex network that combines data from eight sources: Bonneville Power Administration power outages, Bonneville Power Administration transmission lines, weather, weather forecast, lightning, land cover, and social sensor data from two leading platforms, Reddit and Twitter.

## 3 Methodology

**Fig 1:**
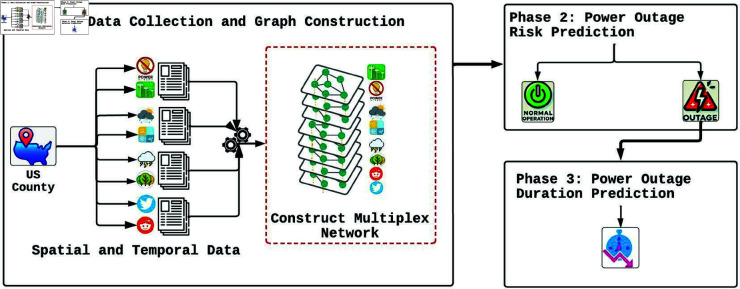
The HMN-RTS framework, a hierarchical spatiotemporal multiplex network, is designed for multi-modal prediction of power outage duration.

This study evaluates the effectiveness of the proposed HMN-RTS hierarchical model, which combines multiplex networks with multi-modal data, in improving the prediction of power outage durations three hours in advance. The HMN-RTS model operates in two distinct stages: first, assessing the risk of power outage occurrence, and second, estimating the severity of the predicted outage by predicting its expected duration, as illustrated in Fig 1. The research is conducted in three main phases: (1) data collection and the construction of spatiotemporal graphs, (2) the development of a spatiotemporal multiplex network for outage risk estimation, and (3) the creation of a model to predict the duration of anticipated outages. Each component of this hierarchical framework is described in detail in the following subsections.

### 3.1 Data collection

Previous studies have demonstrated the advantages of integrating data from various sources to improve the prediction of the severity of power outages [23]. As a first step, we identify and collect key factors essential for predicting outage duration. These factors include power outage records, weather data, weather forecast data, lightning data, land cover information, transmission line data, and social sensor data from two leading platforms: Twitter (now known as X) and Reddit. This subsection outlines in detail the data collection process.

1. **Power outages:** This study focuses on the U.S. Pacific Northwest region. We obtain data on transmission service power outages spanning over 15,000 circuit miles from January 1, 2021, to December 31, 2022. The data is sourced from the Footnotes are not allowed in text, hence we have captured the text as a parenthetical statement, please check and verify.Bonneville Power Administration (BPA), a federal agency operating in the Pacific Northwest (https://www.bpa.gov/). BPA publicly reports all power outages, regardless of the cause. However, this study specifically targets weather-related outages, such as those caused by ice or lightning. To identify these outages, we filtered the data and mapped them to their respective counties and states using a dictionary that links each county to its Federal Information Processing Standards (FIPS) code (https://www.nist.gov/itl/fips-general-information). This process resulted in 2,411 weather-related outages.
2. **Weather:** We obtain historical weather data from Automated Surface Observing Systems (ASOS) (https://mesonet.agron.iastate.edu/request/download.phtml) stations between January 2021 and December 2022 [26]. These stations, situated at airports, are equipped with sensors to monitor various parameters, including temperature, pressure, wind, and visibility obstructions. Each station is mapped to its corresponding county and state using latitude and longitude. This process results in approximately 39 million weather observations collected across five states. The weather dataset includes features such as humidity, dew point temperature, pressure, precipitation, air temperature, wind speed, wind direction, wind gust, apparent temperature, and ice accretion over 6 hours.
3. **Land cover:** Rapidly growing or falling trees can pose a threat to power lines, causing damage and disruptions. To mitigate this risk, we use land cover data from the National Historical Geographic Information System (NHGIS) [49]. This dataset includes land cover classifications such as mixed, deciduous, and evergreen forests, sourced from the National Land Cover Database (NLCD). To integrate this data with other information, we utilize the GISJOIN identifier (https://www.nhgis.org/geographic-crosswalks) provided by [49], which links counties and states using their corresponding FIPS codes.
4. **Lightning:** Lightning strikes during storms can damage utility assets and lead to faults. While the ASOS database does not include data from the National Oceanic and Atmospheric Administration (NOAA) (https://www.ncei.noaa.gov), we rely on NOAA’s publicly available database for information specific to the selected counties and time frame. This database provides daily counts of lightning strikes within a 0.1-degree grid cell, along with the latitude and longitude of each strike. Using this geographic information, we map lightning strike events to their corresponding counties and states. Our analysis focuses on lightning activity during power outages that occurred between January 2021 and December 2022, resulting in the identification of 7,015 lightning strikes in the five states.
5. **Forecast:** The ASOS database lacks forecast data. To obtain this, we utilize the OpenMeteo API, which provides open-source weather forecasts sourced from national weather services (https://open-meteo.com/). Open-Meteo offers weather models with a resolution of 11 km and regional models with resolutions of 1.5 km. The database supplies hourly data for various weather features, including temperature, relative humidity, and wind direction. By specifying the latitudes and longitudes of the counties, we can gather forecast data for each county based on its proximity to the nearest station. Our analysis has focused on studying forecast data during power outages from January 2021 to December 2022, resulting in the collection of 3,959,520 observations across five states. The forecast dataset includes features such as temperature, relative humidity, dewpoint, precipitation, wind speed, and wind direction.
6. **Transmission lines:** This study emphasizes transmission line outages instead of distribution line outages. To support this focus, we gather transmission line data from the Bonneville Power Administration (BPA) service area in the U.S. Pacific Northwest (https://www.bpa.gov/). This information is sourced from the BPA map, which encompasses over 15,000 miles of transmission lines.
7. **Social sensor:** From January 2021 to December 2022, we collected social media data using keywords related to weather and power outages, such as “blackout,” “outage,” “power outage,” and “storm,” from January 2021 to December 2022 [50].
  - **Twitter, “now known as X”**: Initially, we gather tweets using the snscrape tool, a Python package designed for scraping historical tweets. We target tweets within a 10-mile radius of specific geographic coordinates (latitude and longitude) to ensure we capture a diverse range of posts from the neighborhood. This process results in the collection of 8.5 million relevant tweets related to weather and power outage events. In compliance with Twitter’s Terms of Service, we only share Tweet IDs and associated embeddings, and we do not distribute the content of tweets. The Tweet IDs and embeddings used in this study can be found in the GitHub (https://github.com/RAljurbua/HMN-RTS).
  - **Reddit**: We use the Reddit API to gather Reddit posts from county-specific subreddits. After collecting all posts and comments from these subreddits, we filtered them based on keywords related to weather and power outages. As a result, we obtained 353,421 posts, with 95,144 remaining after applying the keyword filtering and selection criteria. In compliance with Reddit’s Terms of Service, we only distribute Reddit IDs and embeddings, not the content of the posts. The Reddit IDs and embeddings used in this study are also available on the GitHub (https://github.com/RAljurbua/HMN-RTS).

### 3.2 Modeling of the spatiotemporal multiplex network

We construct a Hierarchical Spatiotemporal Multiplex Network. Let *G* represent a spatiotemporal multiplex network defined as *G*(*V*,*E*,*L*,*T*), where: $V=\{v_{1},v_{2},...,v_{n}\}$ indicates the set of vertices (counties), $E=\{e_{1},e_{2},...,e_{m}\}$ indicates the set of edges, $L=\{l_{1},l_{2},l_{3},l_{4},l_{5},l_{6},l_{7},l_{8}\}$ indicates the set of layers, and $T=\{t_{1},t_{2},...,t_{k}\}$ indicates a set of time steps. The edges *E* within each layer *L* represent a distinct type of relationship among the vertices *V*. These relationships are defined as follows:

1. **Transmission lines layer:** In layer *l*_1_, two counties $\left(u_{l_{1}},v_{l_{1}}\right)$ are connected if they share a common transmission line. The weight of the edge reflects the number of transmission lines shared between these counties.
2. **Power outage layer:** In layer *l*_2_, two counties $\left(u_{l_{2}},v_{l_{2}}\right)$ are connected if they both experience a power outage on the same date. The edge weight indicates the number of power outages shared between these counties.
3. **Weather layer:** In certain instances, power outages are strongly influenced by weather conditions. Thus, in layer *l*_3_, we connect nodes representing two counties $\left(u_{l_{3}},v_{l_{3}}\right)$ if they exhibit similar weather characteristics. To determine this, we calculate the Euclidean distance between each pair of vertices to capture the similarity in the feature space. Euclidean distance, as a geometric measure of closeness in high-dimensional feature space, provides an interpretable means of quantifying environmental resemblance in weather which can correspond to comparable stress patterns on power systems.
4. **Lightning layer:** In layer *l*_4_, two counties $\left(u_{l_{4}},v_{l_{4}}\right)$ are connected if they both report a lightning strike on the same day. The weight of the edge reflects the number of lightning strikes shared between these counties.
5. **Land cover layer:** In layer *l*_5_, two counties $\left(u_{l_{5}},v_{l_{5}}\right)$ are connected if they share similar land cover characteristics. To determine this, we compute the Euclidean distance between the vertices. Euclidean distance offers an interpretable way to measure environmental similarity in land-cover features which may reflect similar impacts on power systems.
6. **Forecast layer**: In layer *l*_6_, vertices $\left(u_{l_{6}},v_{l_{6}}\right)$ are connected if their weather properties are similar. This is because weather conditions play a significant role in power outages. The similarity between the weather attributes of the two vertices is measured using the Euclidean distance.
7. **Social sensor layers (Twitter and Reddit):** In layers *l*_7_ and *l*_8_, two counties are connected if they both report social media activities during a power outage. The edge weight indicates the number of shared social media posts, such as Tweets or Reddit posts, between the counties.

This graph is used as input for the HMN-RTS model. At the end of each day, a new snapshot of the multiplex graph, timestamped with that day’s data, is added to capture the interdependencies between counties. The model is trained using this snapshot, generating embeddings for the county nodes through the proposed method described in detail in the following subsection. The goal is to predict whether a county will experience a power outage. If an outage occurs, the model forecasts its severity by predicting the duration. This duration prediction is based on county node embeddings and social media data (from Twitter and Reddit) related to weather conditions and power outages.

### 3.3 Proposed model: Hierarchical Spatiotemporal Multiplex Network (HMN-RTS)

This study explores the potential of a hierarchical multiplex network combined with multi-modal data to improve the prediction of disruption severity up to three hours in advance. The approach begins by analyzing an annotated dataset to detect weather-related power outages, acknowledging the variation in outage frequency across states and counties. Each data entry is labeled as one if a power outage occurs within a county during a specified time and as zero otherwise. Subsequently, to estimate outage duration, the outages are classified into five distinct classes, as detailed in Table 1. The proposed model operates in two stages: (1) predicting the occurrence of power outages, followed by (2) predicting outage severity by classifying the duration of the disruptions.

**Table 1:** The distribution of power forced outage durations in the BPA service territory during 2021-2022 is divided into five distinct duration classes.

Class	Duration	Percentage
Class 1	Less than 30 min	63%
Class 2	30 min to 1 hour	1%
Class 3	1 to 3 hours	8%
Class 4	3 to 6 hours	6%
Class 5	Greater than 6 hours	22%

#### 3.3.1 Power outage risk prediction.

The first stage of the HMN-RTS architecture focuses on predicting the occurrence of power outages. The model leverages the multiplex snapshot to create county-level node embeddings using a modified version of node2vec [51] that integrates multiple graph layers. To construct embeddings, the model performs biased random walks over the multiplex graph, allowing inter-layer jumps based on transition probabilities:

$$\pi (v_{i+1}|v_{i},l_{i})\propto \left\{ \begin{array}{cc} \alpha \cdot w^{\left(l_{i}\right)}(v_{i},v_{i+1}) & \text{if }v_{i+1}\in N^{\left(l_{i}\right)}(v_{i})\\ \beta \cdot w^{\left(l^{\prime }\right)}(v_{i},v_{i+1}) & \text{if }l^{\prime }\neq l_{i} \end{array}\right.$$ (1)

α (alpha) and β (beta) are hyperparameters that control the behavior of the random walk across the multiplex graph. These sampled walks are then used to optimize the skip-gram objective for node embeddings:

$$\max_{f}\sum_{u\in V}\sum_{v\in N_{S}(u)}\log\mathrm{Pr}(v|f(u))$$ (2)

$$\mathrm{Pr}(v|f(u))=\frac{\exp(f(v{)}^{⊤}f(u))}{\sum_{w\in V}\exp(f(w{)}^{⊤}f(u))}$$ (3)

Here, $f(u)\in {\mathbb{R}}^{d}$ is the learned embedding of node *u*, and *N*_*S*_(*u*) denotes the sampled context nodes from walks starting at *u*. The model maximizes the likelihood of observing context nodes given the embedding of a source node.

These embeddings are combined with weather data from ASOS and forecast data from OpenMeto for the current day and fed into a neural network model comprising three fully connected layers, with dropout layers applied between them to reduce the risk of overfitting. Due to the dataset’s significant imbalance, fewer than two percent of instances are classified as outages. To address this issue during training, we employ the Synthetic Minority Oversampling Technique (SMOTE) [52]. Binary cross-entropy serves as the loss function for training this component of the HMN-RTS architecture, focusing on isolating instances where the model accurately predicts outages:

$$\text{Loss}(\hat{y},y)=-\frac{1}{N}\sum_{i=1}^{N}\left[y_{i}\log({\hat{y}}_{i})+(1-y_{i})\log(1-{\hat{y}}_{i})\right]$$ (4)

Where ${\hat{y}}_{i}$ is the predicted probability of an outage, $y_{i}\in \{0,1\}$ is the true label for the *i*-th sample, and *N* represents the total number of samples in the dataset.

#### 3.3.2 Power outage duration prediction.

Given the high-frequency nature of ASOS weather data, missing values are inevitable. These can be addressed through various techniques such as removal, imputation, or mean assignment. However, removing missing values may significantly reduce the dataset and risk losing valuable information, while imputation at the county level may introduce bias. To mitigate these issues, we compute the daily average and standard deviation for each weather feature per county. This aggregation strategy reduces the impact of missing data while preserving meaningful patterns.

After predicting a power outage for a county, the next step focuses on forecasting the outage duration three hours in advance using multi-class classification. To achieve this, we collect weather and forecast data at 30-minute intervals within each county. Since counties may have multiple stations and numerous readings per station, we aggregate the data by calculating the mean and standard deviation of each feature, resulting in a normalized and comprehensive representation of local weather conditions.

Next, we collect all Reddit posts from the U.S. Pacific Northwest region and utilize BERT [53] to extract 768-dimensional embeddings for each post. To handle intervals with multiple posts, we apply max pooling, resulting in a single representative vector for each interval. Similarly, for Twitter data, we collect all tweets posted within 30-minute intervals across the U.S. Pacific Northwest and generate 768-dimensional embeddings for each tweet using BERTweet [54]. Max pooling is again used to aggregate these embeddings into a single vector per interval. This method captures the most prominent patterns from social media, offering the model a compact yet informative representation for each 30-minute period. Finally, we concatenate the weather data, forecast data, aggregated Reddit embeddings, Twitter embeddings, and multiplex model embeddings to create a unified input dataset.

The distribution of power outage durations is divided into five distinct duration classes, as outlined in Table 1. We utilize a Bidirectional Long Short-Term Memory (Bi-LSTM). To construct the input for the Bi-LSTM model, we concatenate features from multiple modalities at each time step *t*, forming a unified vector:


$$X_{t}=\;\mu_{t}^{\left(w\right)}\;\|\;\sigma_{t}^{\left(w\right)}\;\|\;\mu_{t}^{\left(f\right)}\;\|\;\sigma_{t}^{\left(f\right)}\;\;\|\;\text{MaxPool}({\text{BERT}}_{\text{Reddit},t})\;\|\;\text{MaxPool}({\text{BERTweet}}_{t})\;\|\;{\text{Node2vec}}_{\text{county}}$$

In this representation, $\mu_{t}^{\left(w\right)}$ and $\sigma_{t}^{\left(w\right)}$ correspond to the mean and standard deviation of the weather features during interval *t*, while $\mu_{t}^{\left(f\right)}$ and $\sigma_{t}^{\left(f\right)}$ denote the mean and standard deviation of forecast features for the same interval. The terms $\text{MaxPool}({\text{BERT}}_{\text{Reddit},t})$ and $\text{MaxPool}({\text{BERTweet}}_{t})$ refer to the max-pooled BERT and BERTweet embeddings, respectively, derived from all Reddit posts and tweets observed within the 30-minute window *t*. Lastly, ${\text{Node2vec}}_{\text{county}}$ represents the county-level structural embedding generated from the multiplex graph using a modified node2vec approach. This concatenated vector captures a comprehensive snapshot of environmental, social, and structural factors, serving as the input to the Bi-LSTM for outage severity classification. The model consists of nine layers with dropout layers applied between them to prevent overfitting. The model is compiled using the Adam optimizer [55] with a learning rate of 0.0001. It is trained for 100 epochs with a batch size of 16. The model is trained using a multiclass categorical cross-entropy loss function, which optimizes its ability to predict outage durations across the predefined classes. The categorical cross-entropy loss evaluates the disparity between the predicted probability distribution $\hat{y}$ and the true labels *y*, ensuring effective model performance.

$$\text{Loss}(\hat{y},y)=-\frac{1}{N}\sum_{i=1}^{N}\sum_{j=1}^{C}y_{ij}\log({\hat{y}}_{ij})$$ (5)

Here, ${\hat{y}}_{ij}$ represents the predicted probability that the *i*th sample belongs to the *j*th class. Similarly, *y*_*ij*_ represents the actual label for the *i*th sample in the *j*th class, which takes a value of 1 if the *i*th sample belongs to the *j*th class, and 0 otherwise. *N* denotes the total number of samples in the dataset, while *C* represents the total number of classes.

## 4 Experimental setup

This study evaluates whether a multiplex network consisting of eight layers, combined with a multi-modal data approach, can enhance the early classification of disruption severity into one of five categories (ranging from short to very long duration). The training period is from January 1, 2022, to June 30, 2022; the validation period is from July 1, 2022, to July 31, 2022; and the testing period is from August 1, 2022, to December 31, 2022. We perform machine-learning experiments to predict the duration of power outages using a supervised machine-learning approach.

We compare the HMN-RTS model with the Long Short-Term Memory (LSTM) and Gated Recurrent Units (GRUs) models. In these approaches, text embeddings are generated using LSTM and GRU, and they do not incorporate the network structure. We further compare the HMN-RTS model with the Reddit and Twitter Multiplex Network (RTMNO) model [23], which is a non-hierarchical approach that does not utilize the capabilities of BERT and is based on a neural network architecture. Additionally, we experiment with a one-layer version, the Hierarchical One-Layer Network Model (HON-RTS). To evaluate the model’s performance, we choose metrics suitable for a power system. Since our classification task consists of five classes, we apply macro-averaging, where each class is given equal importance regardless of its frequency. We calculate macro precision and recall to evaluate the rates of false positives and false negatives. Additionally, we assess the model’s performance on the test set using the macro F1 scores for each class. If *C* denotes the number of classes, the macro F1 score can be defined as:

$$\text{Macro F1}=\frac{1}{C}\sum_{i=1}^{C}2\cdot \frac{{\text{precision}}_{i}\cdot {\text{recall}}_{i}}{{\text{precision}}_{i}+{\text{recall}}_{i}}$$ (6)

## 5 Hierarchical spatiotemporal multiplex network analysis

This section provides the findings from analyzing the topological structure of the weighted multiplex networks we constructed. To gain an in-depth understanding of these networks, we analyze several centrality measures, including Degree Centrality (*DC*), Closeness Centrality (*CC*), Eigenvector Centrality (*EC*), Clustering Coefficient (*CF*), and Square Clustering (*SCF*), in order to investigate the network’s structural characteristics. We report the average values for each of these measures.

Degree Centrality (*DC*) assesses a node’s connectivity by counting the number of edges it is linked to. It offers an understanding of the node’s importance and potential to function as a central hub within the network. Nodes with higher degree centrality are regarded as more central due to their greater number of connections compared to other nodes. It is calculated using the following formula:

$$C_{D}(G)=\frac{\sum_{i=1}^{\left|V\right|}[C_{D}(v*)-C_{D}(v_{i})]}{|V{|}^{2}-3|V|+2},$$ (7)

where, *v* denotes a vertex in the graph *G*. In contrast, Closeness Centrality (*CC*) indicates a node’s proximity to all other nodes in the network. A node with the shortest total distance to all other nodes possesses high Closeness Centrality, making it a crucial node for efficiently disseminating information. The Closeness Centrality can be computed using the following formula:

$$C(v)=\frac{N-1}{\sum_{u}d(u,v)},$$ (8)

where *N* represents the total number of nodes in the graph, and *d*(*u*,*v*) denotes the distance between nodes *u* and *v*. Eigenvector Centrality (EC) considers the importance of a node’s neighbors, evaluating its centrality based not only on its connections but also on the centralities of the nodes it is connected to. The following equation gives the formula for calculating *EC*:

$$x_{v}=\frac{1}{\lambda }\sum_{u\in M(v)}x_{u}=\frac{1}{\lambda }\sum_{u\in G}a_{v,u}x_{u},$$ (9)

where, $A=(a_{v,u})$ represents the adjacency matrix of the graph *G*, *M*(*v*) denotes the set of neighbors of node *v*, and λ is a constant. We also compute the average Clustering Coefficient (CF), which reflects the average number of edges between the neighbors of all nodes. The average clustering coefficients for all vertices *v* are calculated as follows:

$$\bar{C}=\frac{1}{n}\sum_{i=1}^{n}C_{i},$$ (10)

Lastly, Square Clustering (SCF) builds on the traditional Clustering Coefficient by considering the probability that two adjacent nodes have a mutual neighbor that is not directly connected to the original node, thus creating a square-like connection [56]. The SCF can be calculated using the following formula:

$$C(v)=\frac{\sum_{u=1}^{k_{v}}\sum_{w=u+1}^{k_{v}}q_{v}(u,w)}{\sum_{u=1}^{k_{v}}\sum_{w=u+1}^{k_{v}}[a_{v}(u,w)+q_{v}(u,w)]},$$ (11)

where, $q_{v}(u,w)$ represents the number of shared neighbors between *u* and *w*, excluding *v*. Table 2 presents the detailed network analysis. The graph consists of 1864 nodes and 10,058,477 edges. An important feature of a multiplex network is the presence of coupled edges (couplingE), which signify the movement of nodes between adjacent layers [57]. Networks with more coupled edges typically have more dense and robust connectivity than those with few or no coupled edges. In this network, there are 13,048 coupling edges. The graph contains 233 unique nodes, with an average Degree Centrality (*DC*) of 5.79, indicating a moderate level of connectivity among the nodes. The average Closeness Centrality (*CC*) is 0.40, suggesting that, on average, the nodes are reasonably close to each other within the network. The Eigenvector Centrality (*EC*) has a low average of 0.01, indicating that the network centrality is not heavily influenced by a few key nodes. The average Clustering Coefficient (*CF*) of 0.80 indicates that, on average, nodes have a high tendency to form triangles. Lastly, the average Square Clustering Factor (*SCF*) is 0.29, reflecting the moderate likelihood of nodes forming square-shaped connections with their neighbors.

**Table 2:** Multiplex network topological structure. *L*= number of layer, *V*= number of nodes, *E*= number of total edges, couplingE= number of coupling edges, *avg*(*DC*)=average Degree Centrality, *avg*(*CC*)=average Closeness Centrality, *avg*(*EC*)=average Eigenvector Centrality, *avg*(*CF*) Clustering Coefficient, *avg*(*SCF*)=average Square Clustering.

L	V	E	couplingE	avg(DC)	avg(CC)	avg(EC)	avg(CF)	avg(SCF)
8	1864	10*M*	13048	5.79	0.40	0.01	0.80	0.29

## 6 Results and discussion

The results of different evaluation metrics for LSTM, GRUs, RTMNO, HON-RTS, and the proposed Hierarchical Multiplex Network model (HMN-RTS) are shown in Table 3. We observe that both LSTM and GRU models achieve similar macro F1 scores of 0.16 and 0.17, respectively. The limited performance can be attributed to the lack of network structure and advanced pre-trained language models. Although the HON-RTS model shows improvements over the non-hierarchical Reddit and Twitter Multiplex Network (RTMNO), it still falls short of the HMN-RTS model. Specifically, the HON-RTS achieves a macro F1 score of 0.73, while the HMN-RTS model outperforms it with a macro F1 score of 0.79. These results indicate that the hierarchical one-layer structure does not fully capitalize on the advantages provided by the multiplex network architecture. In contrast, the improved performance of HMN-RTS highlights the critical importance of leveraging multiplex network capabilities and the multi-modal approach. By integrating multiple layers and their interactions, HMN-RTS effectively captures complex correlations that single-layer approaches fail to represent. This comprehensive representation leads to a notable improvement in predictive performance, particularly in modeling outage duration across five distinct classes. The results underscore how multiplex network integration is beneficial for accurately predicting the severity of power outages.

**Table 3:** A comparison of macro precision, macro recall, and macro F1 score is conducted across various models, including Long Short-Term Memory (LSTM), Gated Recurrent Units (GRUs), the Reddit and Twitter Multiplex Network (RTMNO), the Hierarchical One-Layer Network Model (HON-RTS), and the Hierarchical Multiplex Network Model (HMN-RTS). The outage duration is categorized into five classes, as outlined in Table 1.

Model	Macro precision	Macro recall	Macro F1 Score
LSTM	0.15	0.20	0.16
GRUs	0.15	0.20	0.17
RTMNO [23]	0.51	0.56	0.53
HON-RTS	0.64	0.84	0.73
HMN-RTS	0.74	0.84	0.79

**Fig 2:**
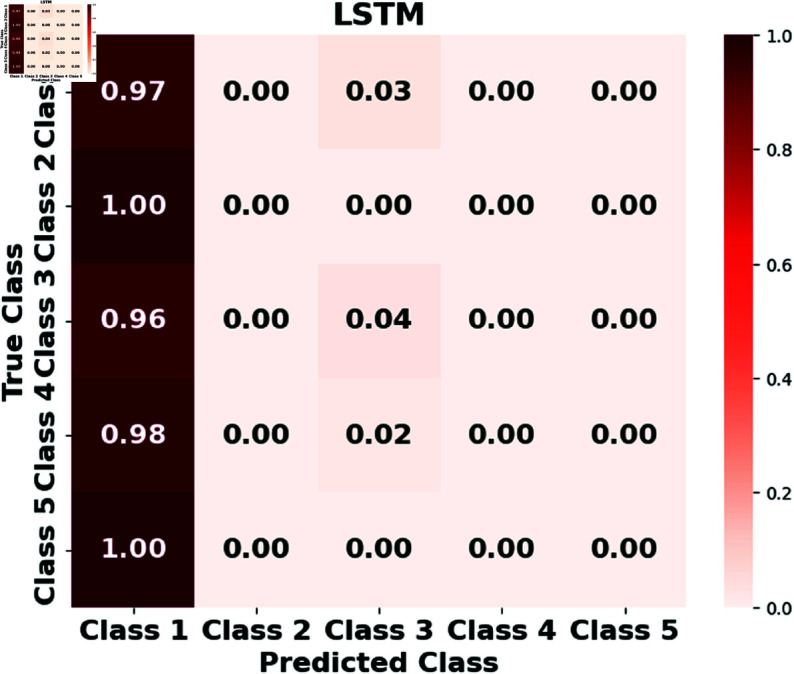
The normalized confusion matrix for the LSTM model illustrates its performance in predicting outage durations.

**Fig 3:**
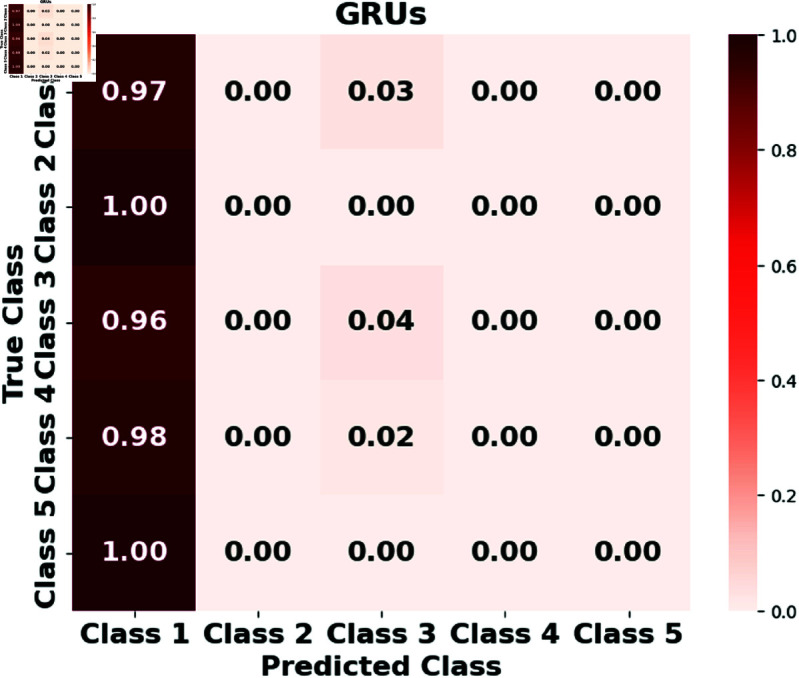
The normalized confusion matrix for the GRUs model illustrates its performance in predicting outage durations.

**Fig 4:**
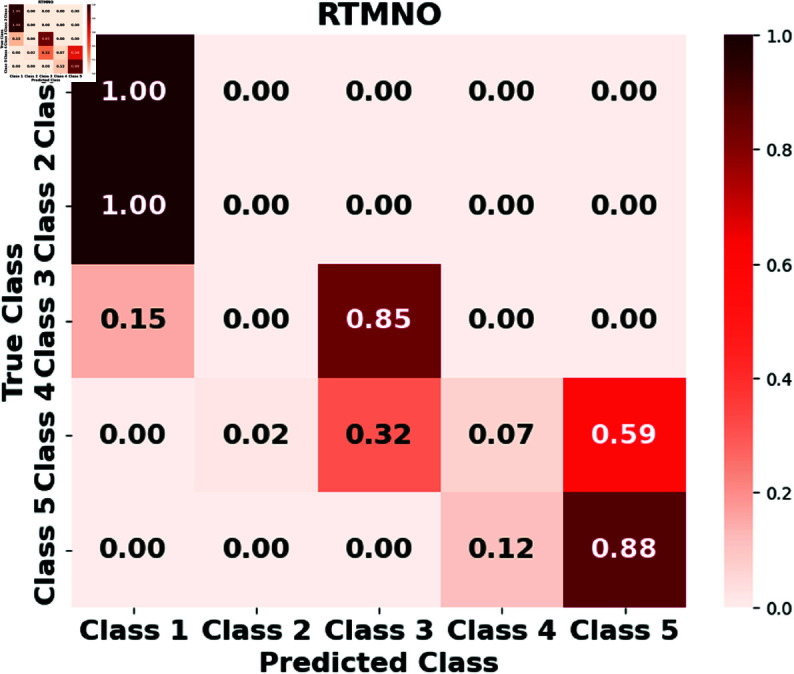
The normalized confusion matrix for the RTMNO model illustrates its performance in predicting outage durations.

**Fig 5:**
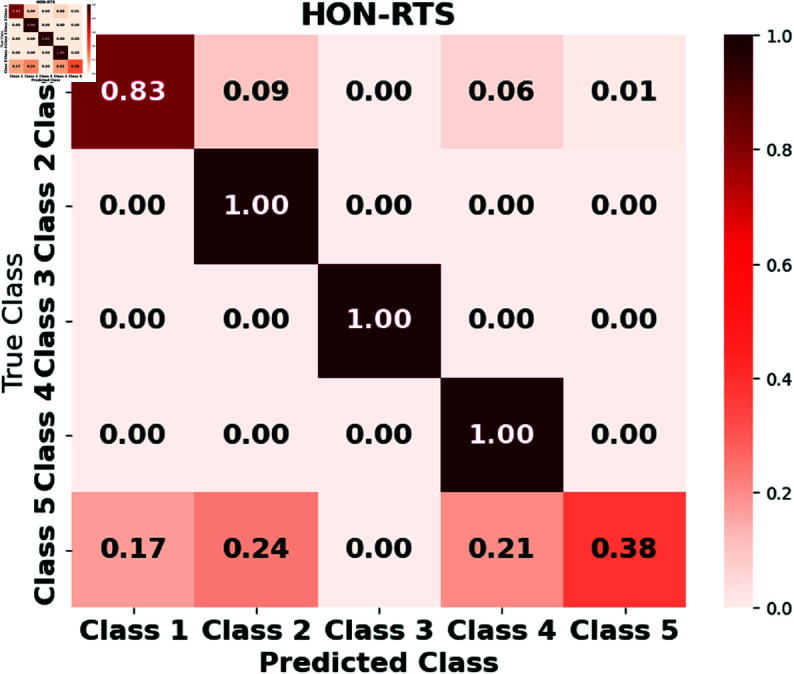
The normalized confusion matrix for the HON-RTS model illustrates its performance in predicting outage durations.

**Fig 6:**
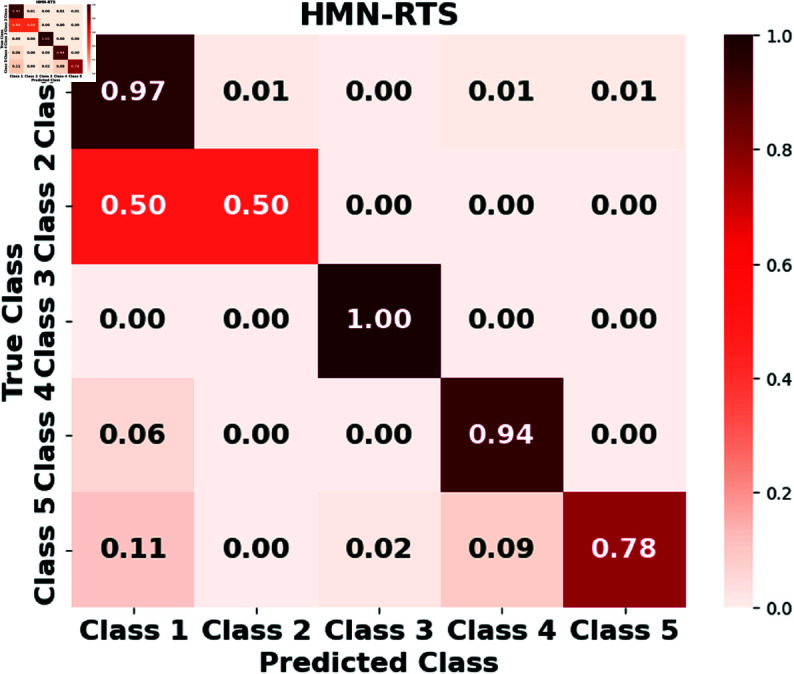
The normalized confusion matrix for the HMN-RTS model illustrates its performance in predicting outage durations.

Furthermore, we assess the performance of the LSTM, GRUs, RTMNO, HON-RTS, and HMN-RTS models using a confusion matrix, which displays the number of correct and incorrect predictions for each class, as shown in Figs 2, 3, 4, 5, and 6. Class 1 represents durations under 30 minutes, Class 2 corresponds to durations between 30 minutes and 1 hour, Class 3 represents durations from 1 to 3 hours, Class 4 represents durations from 3 to 6 hours, and Class 5 is for durations exceeding 6 hours. Notably, the HMN-RTS model shows the best performance in correctly predicting the outage duration classes, achieving higher classification accuracy across most categories. This is reflected in its significantly improved accuracy compared to the other models, especially in terms of reducing false negatives and correctly identifying the most severe outages. In addition, the confusion matrix for the HMN-RTS model reveals that the model performs particularly well in distinguishing short-duration outages (Class 1) from long-duration events (Class 5), which is crucial for prioritizing emergency response. Misclassifications tend to occur between adjacent classes, which is expected in a real-world scenario. Class 2 accounts for only 1% of the data, which likely contributes to the model’s tendency to misclassify it as Class 1. However, due to the short duration associated with Class 2 events, this misclassification is unlikely to have a significant impact on emergency response efforts.

In the second set of experiments, an ablation study is conducted. For this, we develop five distinct versions of HMN-RTS. The first version, HMN-R, is the model that employs only Reddit information, processing only Reddit data. The second version, HMN-T, is the model that utilizes only Twitter data. The third version, HMN-S, is the model that employs only the multiplex network structure. The model processes only multiplex network data. The fourth version, HMN-RT, is the model that combines both Reddit and Twitter data, processing information from both platforms. Lastly, HMN-RTS is the model that integrates Reddit, Twitter, and the multiplex network structure. The performance of these models is summarized in Table 4. The results show a clear trend: the addition of each component incrementally improves the model performance. In particular, the HMN-S model outperforms HMN-R and HMN-T, highlighting the strength of the multiplex network structure itself in capturing relational dependencies. However, the HMN-RTS, which leverages multi-modal data along with the network structure, achieves the best macro F1 score of 0.79, demonstrating the integrated value of combining social context with network structure. These findings emphasize that no single modality is sufficient on its own; instead, the multi-modality of heterogeneous sources through a multiplex network is key to accurately capturing the complex relationships embedded in multi-modal data that influence outage durations.

**Table 4:** The ablation study of the HMN-RTS model investigates the contributions of its individual components. The Multiplex Network Structure includes the layers outlined in Sect 3.2, while Reddit and Twitter represent the activities captured from social sensors.

Settings	Reddit	Twitter	Multiplex Network Structure	Macro precision	Macro recall	Macro F1 Score
HMN-R	✓			0.59	0.81	0.69
HMN-T		✓		0.63	0.81	0.71
HMN-S			✓	0.68	0.79	0.73
HMN-RT	✓	✓		0.66	0.88	0.76
HMN-RTS	✓	✓	✓	0.74	0.84	0.79

**Fig 7:**
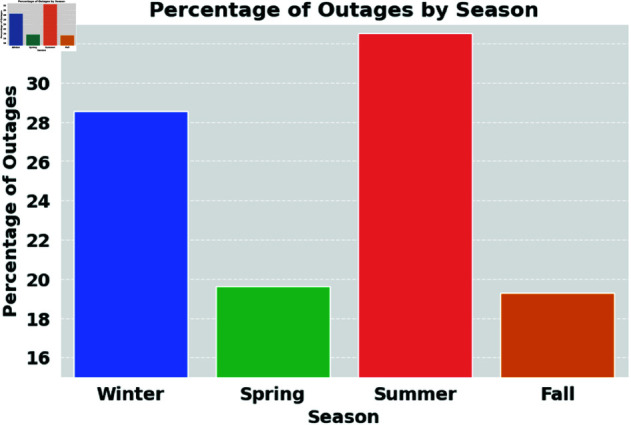
The performance of the HMN-RTS model in the early detection of outages is evaluated using the macro F1 score for a five-class problem, as detailed in Table 1. The X-axis in the corresponding figure shows the number of hours before the power outage at which predictions are made.

To evaluate the robustness of the HMN-RTS model across different time horizons, we conduct a third experiment focused on early-stage predictions. This is particularly valuable in power systems, where earlier forecasts can enable proactive planning and mitigation. Fig 7 illustrates the macro F1 score of the HMN-RTS model for early predictions, with the X-axis representing the number of hours before the outage event. The results show a consistent trend: the macro F1 score improves as the prediction time gets closer to the outage, aligning with expectations that the outcomes become more predictable over shorter horizons. Notably, the HMN-RTS model achieves a high macro F1 score for predictions made up to 6 hours before the outage. Even 12 hours in advance, the model demonstrates solid performance, indicating that patterns start to emerge well ahead of the actual disruption. This early warning capability makes HMN-RTS highly valuable for utilities and emergency response teams that require sufficient lead time to coordinate effective responses.

**Fig 8:**
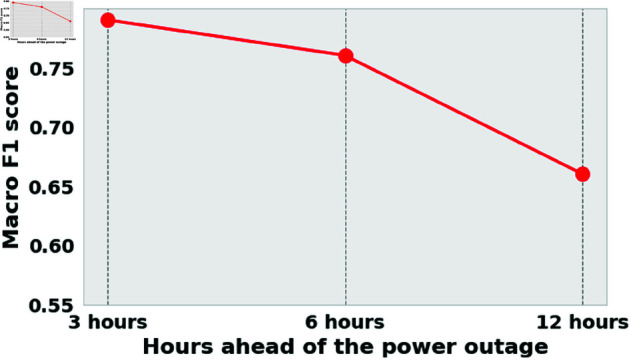
Percentage of power outages by season during 2021–2022.

To assess the robustness of HMN-RTS across different seasonal data distributions, the fourth experiment retrains and tests the HMN-RTS model using data from each season. Fig 8 shows the percentage of power outages by season, highlighting that outages are most frequent in winter and summer. The data reveals that summer and winter experience the highest percentage of outages. This trend suggests that weather-related conditions in winter and summer, such as snowstorms, freezing temperatures in winter, heatwaves, and increased energy demand in summer, contribute to higher outage rates. Given this distribution, this experiment assesses the ability of the HMN-RTS model to predict power outages, with a focus on winter and summer, as these seasons pose the most significant challenges to power grid stability and provide the most valuable insights into outage patterns. Given the limitation of having only two years of data, we split the dataset into training, validation, and test sets, with a 80%-10%-10% ratio. Additionally, to ensure robust model evaluation, we apply 10-fold cross-validation to further assess the Hierarchical Multiplex Network Model (HMN-RTS) performance. Table 5 represents the results of the seasonal experiments. While the HMN-RTS model performs consistently well across all seasons, its performance is further improved with a more homogeneous season-specific dataset, highlighting how seasonal consistency can enhance prediction accuracy. This suggests that while the HMB-RTS model is robust year-round, it particularly excels when the data is seasonally filtered.

**Table 5:** A comparison of the average macro F1 scores for two seasonal models (Winter and Summer) using the Hierarchical Multiplex Network Model (HMN-RTS). Note that the standard deviations in all models are between [0.08,0.1].

Season	Average macro precision	Average macro recall	Average macro F1 Score
Summer	0.89	0.86	0.87
Winter	0.90	0.87	0.88

## 7 Conclusion

Power outages can disrupt daily routines, affecting areas like transportation and communication. Therefore, predicting their severity is essential for efficient planning. This study presents a Hierarchical Multiplex Network (HMN-RTS) approach at the county level, specifically for the U.S. Pacific Northwest. The method aims to predict the occurrence and duration of weather-related power outages. The HMN-RTS model forecasts outages and their severity over multiple time horizons and across multiple seasons. We leverage the multi-modal approach by collecting data from multiple sources, including weather data, weather forecast data, lightning data, land cover information, transmission lines, and social sensor data from two leading platforms. We evaluate the effectiveness of the proposed hierarchical multiplex network approach in enhancing prediction performance compared to other models. Our hierarchical spatiotemporal multiplex network improves the accuracy of predictions for three-hour-ahead outage durations. Achieving a macro F1 score of 0.79, this method enables grid operators to promptly implement strategies for mitigating outages.

The limitation of this study is that it relies completely on ASOS weather data, and future work could explore additional sources, such as ERA5, to enhance the environmental context. Studies could consider integrating datasets such as OpenMeteo and ERA5 to strengthen the environmental context. In the case of social media data, user engagement can be influenced by demographic factors such as age, and different subsets of the population may be active on different platforms. As this study focused on two social media sources, it may not fully capture the diversity of public response within the target geographic area. Future research could explore a hierarchical modeling approach that separately predicts outage occurrence and duration, especially within distribution grids, which exhibit distinct patterns and challenges compared to transmission systems.
